# Long-term outcomes of robot-assisted laparoscopic surgery versus conventional laparoscopic surgery for rectal cancer: single-center, retrospective, propensity score analyses

**DOI:** 10.1007/s11701-024-01894-x

**Published:** 2024-04-03

**Authors:** Junichi Mazaki, Tetsuo Ishizaki, Yu Kuboyama, Ryutaro Udo, Tomoya Tago, Kenta Kasahara, Tesshi Yamada, Yuichi Nagakawa

**Affiliations:** Department of Gastrointestinal and Pediatric Surgery, Tokyo Medical University, Tokyo, Japan

**Keywords:** Robot-assisted laparoscopic surgery, Conventional laparoscopic surgery, Rectal cancer, Long-term outcomes, Short-term outcomes, Propensity score

## Abstract

Although the short-term outcomes of robot-assisted laparoscopic surgery (RALS) for rectal cancer are well known, the long-term oncologic outcomes of RALS compared with those of conventional laparoscopic surgery (CLS) are not clear. This study aimed to compare the long-term outcomes of RALS and CLS for rectal cancer using propensity score matching. This retrospective study included 185 patients with stage I–III rectal cancer who underwent radical surgery at our institute between 2010 and 2019. Propensity score analyses were performed with 3-year overall survival (OS) and relapse-free survival (RFS) as the primary endpoints. After case matching, the 3-year OS and 3-year RFS rates were 86.5% and 77.9% in the CLS group and 98.4% and 88.5% in the RALS group, respectively. Although there were no significant differences in OS (*p* = 0.195) or RFS (*p* = 0.518) between the groups, the RALS group had slightly better OS and RFS rates. 3-year cumulative (Cum) local recurrence (LR) and 3-year Cum distant metastasis (DM) were 9.7% and 8.7% in the CLS group and 4.5% and 10.8% in the RALS group, respectively. There were no significant differences in Cum-LR (*p* = 0.225) or Cum-DM (*p* = 0.318) between the groups. RALS is a reasonable surgical treatment option for patients with rectal cancer, with long-term outcomes similar to those of CLS in such patients.

## Introduction

Of the various types of surgery for rectal cancer, minimally invasive surgery (MIS) is increasingly being performed for patients with this condition. Compared to open surgery (OS), MIS for rectal cancer has many advantages owing to the introduction of advanced instruments and evolution of surgical techniques. Evaluation of conventional laparoscopic surgery (CLS) for rectal cancer in randomized clinical trials [1, 2] revealed that it had similar or better short-term and similar long-term oncological outcomes compared to those of OS [3, 4].

One recent study examined the short-term results for rectal cancer of the latest approach, robot-assisted laparoscopic surgery (RALS). The ROLARR randomized clinical trial compared RALS with CLS for rectal cancer and tried to evaluate procedural superiority in terms of conversion rates; however, the results were not definitive [5]. Nonetheless, favorable safety and technical feasibility-related outcomes of RALS for rectal cancer have been reported in several retrospective case–control studies [6–10] and small randomized clinical trials [11–13]. However, evidence regarding the long-term outcomes of RALS remains scarce [14–16]. Furthermore, only one study has reported long-term RALS results using propensity score matching [17]. Our hospital was one of the first to introduce robotic surgical systems in Japan.

Propensity score analysis is used to minimize selection bias in retrospective studies and increase the strength of the evidence. Therefore, this study used propensity scores to compare the long-term results of RALS and CLS for rectal cancer.

## Methods

### Patients

In total, 311 patients who underwent radical surgery for stage I–III rectal cancer at our institute between 2010 and 2019 were retrospectively reviewed. Of these patients, we excluded those for whom we did not have sufficient data. Finally, we divided the remaining 185 patients into the CLS and RALS groups according to the surgical procedure they underwent. Based on the Union for International Cancer Control (UICC) Tumor-Node-Metastasis (TNM) Classification, 8th edition, 23 patients who were diagnosed with cN-positive lower rectal cancer underwent surgery with lateral lymph node dissection (LLND) after receiving neoadjuvant chemotherapy (NAC). This study was reviewed and approved by the institutional review board of our institute, and the requirement for written informed consent was waived due to the retrospective design.

### Surgical procedures

All the patients underwent gross curative RALS or CLS with lymph node dissection. Using the Da Vinci Si or Xi Surgical System (Intuitive Surgical, Sunnyvale, CA, USA) as a six- or five-port system, two certified surgeons performed RALS. Five ports were generally placed when using the Xi Surgical System; six ports were placed in some cases when suction was needed for excessive fluid, such as for patients who underwent NAC. Six ports are generally placed when using the Si Surgical System. The patients were placed in a lithotomy position with the head down at 15° and the right side down at 15°. All procedures were performed in the colonic or pelvic phases. Inferior mesenteric artery, vein ligation, and left-sigmoid mesocolon mobilization were performed in the colonic phase, while pelvic dissection using total mesorectal excision (TME) or tumor-specific mesorectal excision (TSME) was performed during the pelvic phase. During TSME for tumors of the upper or mid-rectum, the distal rectum was divided > 3 cm below the lower border of the tumor. In contrast, during TME for tumors of the lower rectum, the distal rectum was divided > 2 cm below the lower border of the tumor. The distal rectum was intracorporeally divided using a linear stapler. Bowel continuity was restored using the intracorporeal double-stapling technique with a circular stapler or transanal hand-sewn suture in cases of intersphincteric resection (ISR). En bloc regional lymphadenectomy was performed for all patients who underwent standard curative resection. When the short diameter of the mesenteric or lateral lymph node was > 7 mm on preoperative magnetic resonance imaging (MRI), LLND was performed after NAC. LLND was performed around the common and internal iliac vessels, obturator space, and in the fat tissue outside the pelvic plexus while preserving all autonomic nerves. Diverting ileostomy was performed, if necessary, during low anterior resection and intersphincteric resection.

### Follow-up

The median follow-up period was 69.9 months (range, 1.1–147.9 months). Patients with stage I–III tumors were followed-up for five years post-operatively. We measured tumor markers every three months for the first two years; thereafter, we measured the tumor markers and performed computed tomography scans every six months for the next three years. If recurrence was suspected, MRI and/or positron emission tomography was used to confirm metastasis. Surgery was considered for patients with a good performance status and resectable recurrence. When the first recurrence occurred, the recurrence site and date were recorded. Local recurrence (LR) was defined as any histological or clinical evidence of tumor regrowth near the primary site. Tumor recurrence at distant sites, such as the liver or lungs, was recorded as distant metastasis (DM). Postoperative urinary function and sexual function, were assessed using the International Prostate Symptom Score (IPSS),

### Statistical analysis

The primary outcomes were the 3-year OS and 3-year relapse-free survival (RFS). We defined OS as the duration between the date of surgery and date of death or end of the observation period. Patients who were alive at the end of the follow-up period were excluded. We defined RFS as the duration between the date of surgery and the date of recurrence or death from the underlying disease. Patients who died from causes other than colorectal cancer were also excluded. Survival outcomes were examined using the Kaplan–Meier method and compared using the log-rank test. Propensity score analyses were performed to adjust for the heterogeneity between the CLS and RALS groups. Propensity scores predicting the probability of receiving CLS or RALS were generated by multivariate logistic regression with the following fourteen covariates: age, sex (female/male), body mass index (BMI), tumor location (lower rectum [0–5 cm]/middle rectum [6–10 cm]/upper rectum [> 10 cm]), neoadjuvant therapy, LLND, pathological T-stage, pathological lymph node metastasis (positive/negative), lymphatic invasion (positive/negative), venous invasion (positive/negative), differentiation (well/others), tumor size, radial margin (positive/negative), anastomotic leakage (yes/no), and postoperative complications (Clavien–Dindo class > 1). Each patient was assigned an estimated propensity score that represented the predicted probability of receiving CLS or RALS. We performed propensity score matching to pair the patients based on similarities in their characteristics. Each patient who underwent CLS was matched to a patient who underwent RALS and had a similar propensity score on the logit scale, with a caliper of 0.2. All statistical analyses were performed using SPSS version 25 (IBM® SPSS® Statistics 25.0 Windows^®^ client version; IBM, Chicago, IL, USA). The level of statistical significance was set at* p* < 0.05.

## Results

### Short-term outcomes

There were 108 and 77 patients in the CLS and RALS groups, respectively. The matched cohort comprised 76 pairs of patients. The characteristics of the patients in the two groups are shown in Table 1.. Patients in the RALS group were more likely to have a longer operative time (*p* = 0.003), shorter length of hospital stay (*p* = 0.022), and IPS (*p* < 0.001) before case matching. No significant differences were observed in covariates or other outcomes. After case matching, the clinical outcomes in terms of the covariates examined were similar between the CLS and RALS groups. Patients in the RALS group were more likely to have a longer operative time (*p* < 0.001) and a lower IPSS (*p* < 0.001) after case matching.

**Table 1:** Characteristics of patients in the CLS and RALS groups and short-term outcomes

	Approach		*p*-value	SMD
	CLS (*n*=108)	RALS (*n*=77)		
Age, y.o.	65 (38–83)	64 (33–82)	0.182	0.227
Sex, *n* (%)				
Female	37 (34.3)	26 (33.8)	1	0.01
Male	71 (65.7)	51 (66.2)		
BMI				
< 25	84 (77.8)	61 (79.2)	0.858	0.035
> 25	24 (22.2)	16 (20.8)		
Location, *n* (%)				
Lower rectum (0–5 cm)	35 (32.4)	19 (24.7)	0.141	0.299
Middle rectum (6–10 cm)	55 (50.9)	36 (46.8)		
Upper rectum (10 cm–)	18 (16.7)	22 (28.6)		
Neo-adjuvant therapy, *n* (%)				
No	99 (91.7)	68 (88.3)	0.462	0.112
Yes	9 (8.3)	9 (11.7)		
Lateral lymph node dissection, *n* (%)				
No	81 (75.0)	62 (80.5)	0.477	0.133
Yes	27 (25.0)	15 (19.5)		
Pathological *T*-stage, *n* (%)				
1	20 (18.5)	19 (24.7)	0.619	0.2
2	23 (21.3)	12 (15.6)		
3	57 (52.8)	39 (50.6)		
4	8 ( 7.4)	7 ( 9.1)		
Pathological *N*-stage, *n* (%)				
Negative	62 (57.4)	41 (53.2)	0.653	0.084
Positive	46 (42.6)	36 (46.8)		
Lymphatic invasion, *n* (%)				
Negative	39 (36.1)	25 (32.5)	0.641	0.077
Positive	69 (63.9)	52 (67.5)		
Venous invasion, *n* (%)				
Negative	26 (24.1)	25 (32.5)	0.244	0.187
Positive	82 (75.9)	52 (67.5)		
Differentiation, *n* (%)				
Well	26 (24.1)	22 (28.6)	0.501	0.102
Others	82 (75.9)	55 (71.4)		
Tumor size, mm	40 (2.7–670)	30 (1–75)	0.073	0.257
RM, *n* (%)				
Negative	102 (94.4)	76 (98.7)	0.242	0.236
Positive	6 ( 5.6)	1 (1.3)		
Postoperative complication, *n* (%) (Clavien–Dindo > 1)				
No	75 (69.4)	53 (68.8)	1	0.013
Yes	33 (30.6)	24 (31.2)		
Anastomotic leakage, *n* (%)				
No	95 (88.0)	67 (87.0)	1	0.029
Yes	13 (12.0)	10 (13.0)		
Operating time, minute	370 (85–891)	464 (188- 965)	0.003	0.47
Intraoperative blood loss, g	51 (1–892)	63 (1–476)	0.41	0.053
Conversion rate, *n* (%)	10 ( 9.3)	4 (5.2)	0.402	0.157
Number of harvested lymph node, *n* (%)	20 (2–96)	20 (3–76)	0.871	0.104
Length of hospital stay, days	17 (8–76)	14 (8–67)	0.022	0.114
Total IPSS scores	6 (1.01)	2 (0.57)	<0.001	4.876

	Approach		*p*-value	SMD
	CLS (*n*=76)	RALS (*n*=76)		
Age, y.o.	63 (39–88)	64 (33–82)	0.7	0.067
Sex, *n* (%)				
Female	29 ( 38.2)	26 (34.2)	0.736	0.082
Male	47 ( 61.8)	50 (65.8)		
BMI				
< 25	66 ( 86.8)	61 (80.3)	0.382	0.178
> 25	10 ( 13.2)	15 (19.7)		
Location, *n* (%)				
Lower rectum (0–5 cm)	20 ( 26.3)	19 (25.0)	0.394	0.22
Middle rectum (6–10 cm)	41 ( 53.9)	35 (46.1)		
Upper rectum (10cm–)	15 ( 19.7)	22 (28.9)		
Neo-adjuvant therapy, *n* (%)				
No	70 ( 92.1)	67 (88.2)	0.588	0.133
Yes	6 ( 7.9)	9 (11.8)		
Lateral lymph node dissection, *n* (%)				
No	63 ( 82.9)	62 (81.6)	1	0.034
Yes	13 ( 17.1)	14 (18.4)		
Pathological *T*-stage, *n* (%)				
1	17 ( 22.4)	19 (25.0)	0.797	0.17
2	17 ( 22.4)	12 (15.8)		
3	35 ( 46.1)	38 (50.0)		
4	7 ( 9.2)	7 ( 9.2)		
Pathological *N*-stage, *n* (%)				
Negative	43 ( 56.6)	41 (53.9)	0.87	0.053
Positive	33 ( 43.4)	35 (46.1)		
Lymphatic invasion, *n* (%)				
Negative	24 (31.6)	25 (32.9)	1	0.028
Positive	52 (68.4)	51 (67.1)		
Venous invasion, *n* (%)				
Negative	22 (28.9)	25 (32.9)	0.726	0.085
Positive	54 (71.1)	51 (67.1)		
Differentiation, *n* (%)				
Well	23 (30.3)	22 (28.9)	1	0.029
Others	53 (69.7)	54 (71.1)		
Tumor size, mm	34 (2.7–128)	31.5 (1–75)	0.5	0.11
RM, *n* (%)				
Negative	76 (100.0)	75 (98.7)	1	0.163
Positive	0 (0.0)	1 (1.3)		
Postoperative complication, *n* (%)(Clavien–Dindo > 1)				
No	51 ( 67.1)	53 (69.7)	0.862	0.057
Yes	25 ( 32.9)	23 (30.3)		
Anastomotic leakage, *n* (%)				
No	67 (88.2)	67 (88.2)	1	<0.001
Yes	9 (11.8)	9 (11.8)		
Operating time, minute	346 (85–872)	457 (188–965)	<0.001	0.621
Intraoperative blood loss, g	50 (1–892)	62 (1–476)	0.901	0.02
Conversion rate, *n* (%)	9 (11.8)	4 (5.3)	0.245	0.237
Number of harvested lymph node, *n* (%)	19 (3–96)	19 (7–76)	0.814	0.038
Length of hospital stay, days	16 (8–66)	14 (8–67)	0.506	0.108
Total IPSS scores	6 (5–8)	2 (1–3)	<0.001	4.809

Data are presented as median (range) or *n* (%).

*BMI* body mass index, *RM* radial margin, *IPSS* international prostate symptom score, *CLS* conventional laparoscopic surgery, *RALS* robot-assisted laparoscopic surgery

### Overall and relapse-free survival

Before case matching, the 3-year OS and 3-year RFS rates were 86.7% and 76.8% in the CLS group and 98.4% and 88.6% in the RALS group, respectively. There were no significant differences in OS (*p* = 0.14) or RFS (*p* = 0.08) between the groups.

**Fig. 1:**
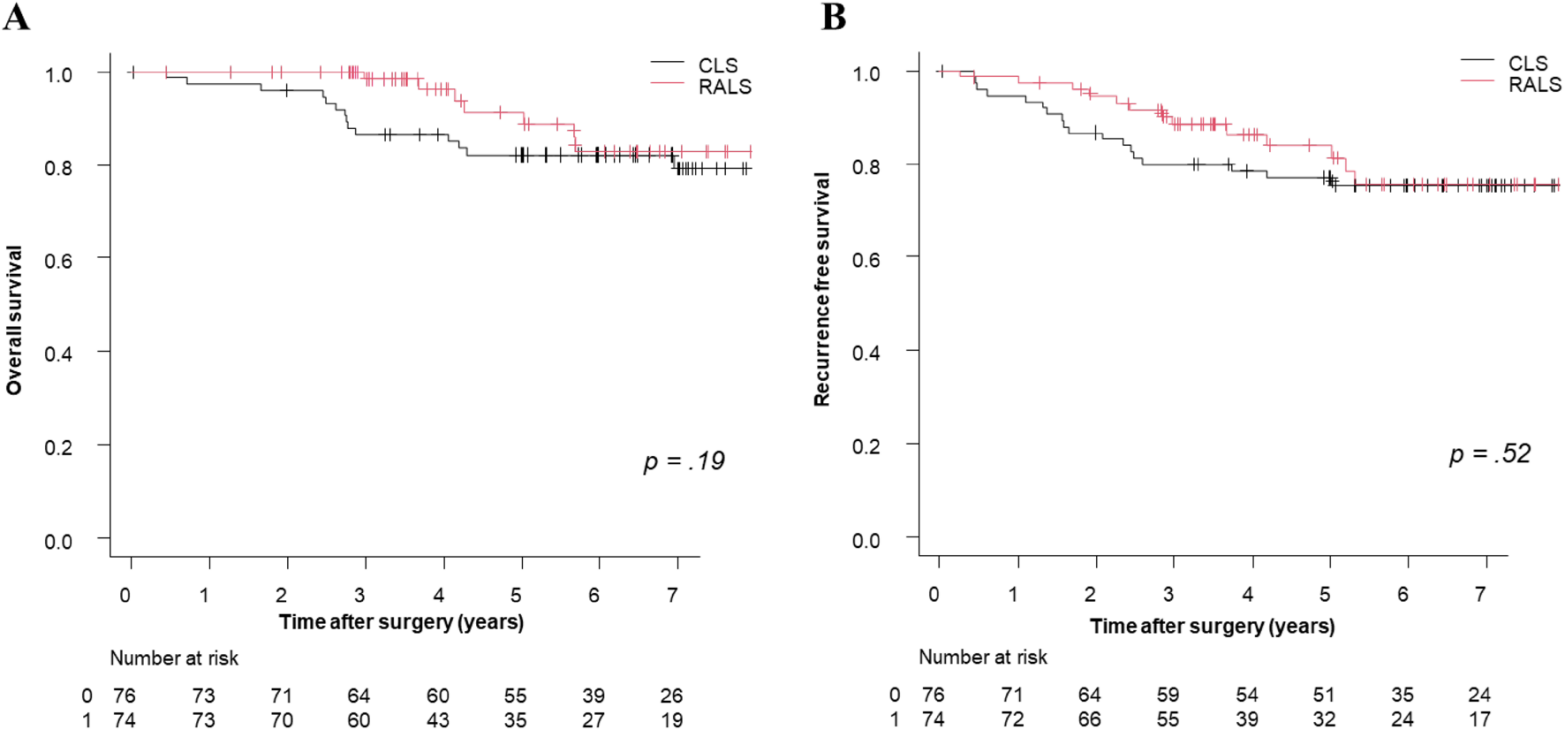
Overall survival and recurrence-free survival rates by surgical procedure in the matched cohort. **A** Overall survival rates, **B** Recurrence-free survival rates

**Fig. 2:**
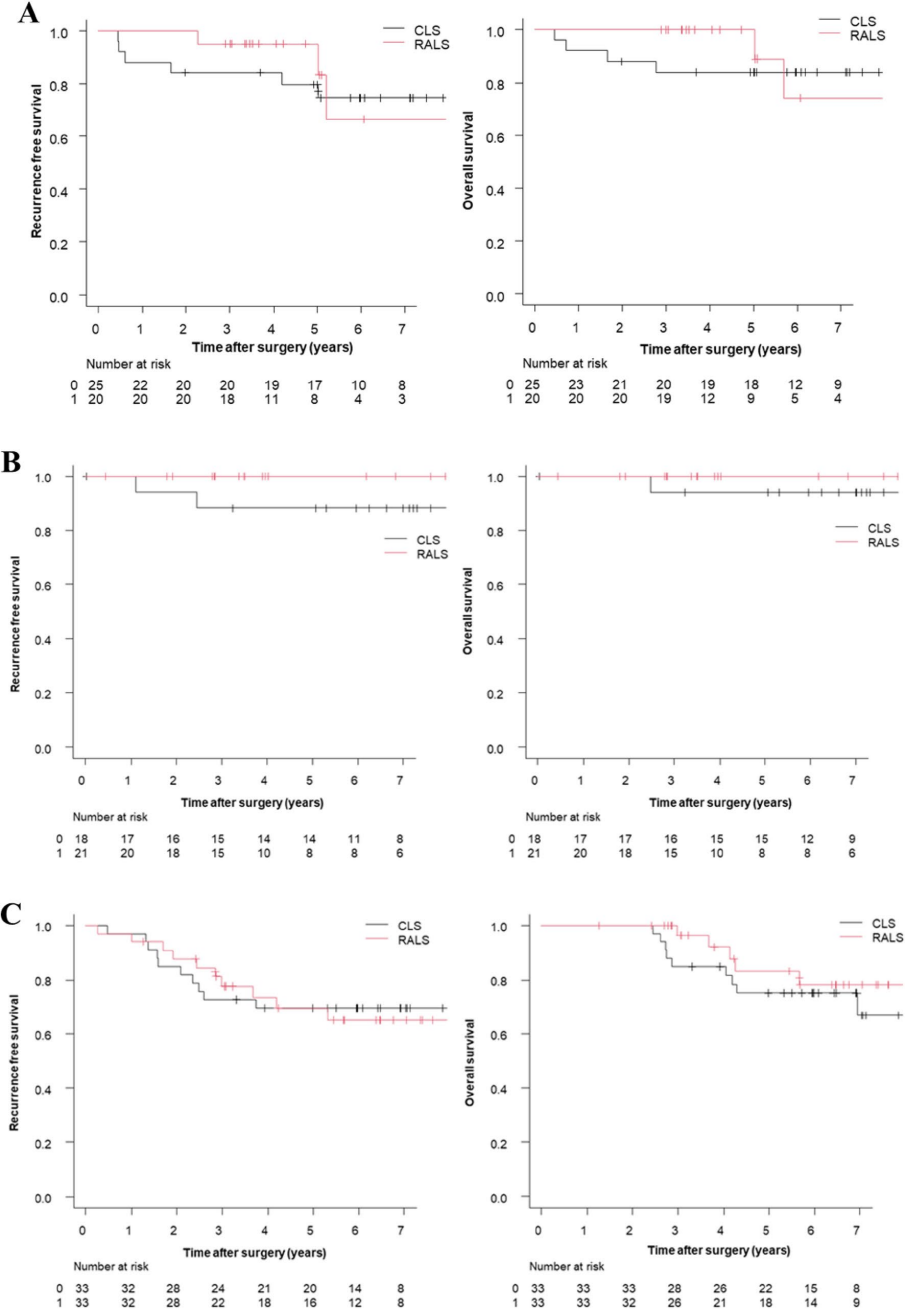
Overall survival and recurrence-free survival rates at each stage in the matched cohort. **A** Overall survival rates and recurrence-free survival rates in stage I rectal cancer, **B** Overall survival rates and recurrence-free survival rates in stage II rectal cancer, **C** Overall survival rates and recurrence-free survival rates in stage III rectal cancer

**Fig. 3:**
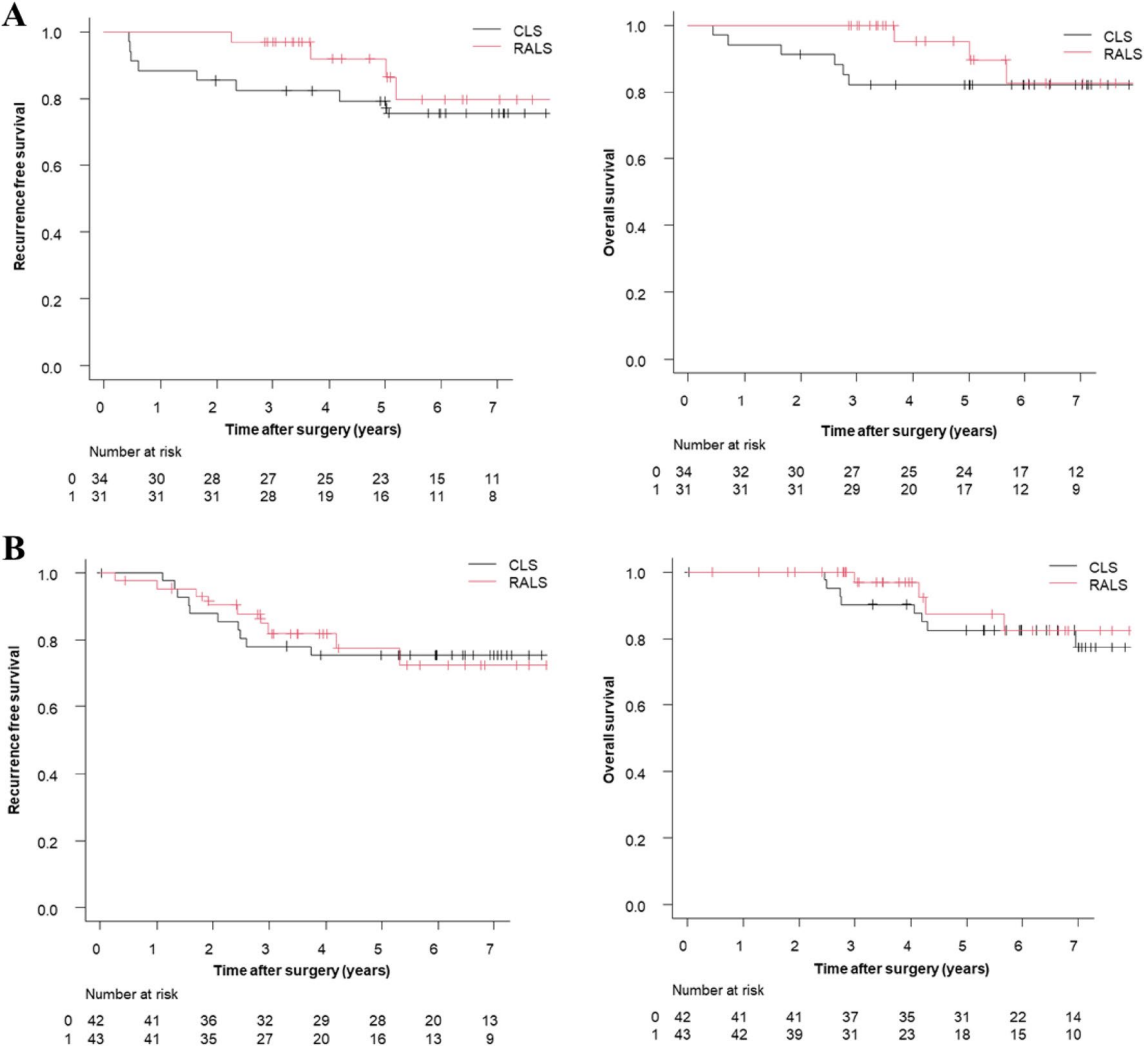
Overall survival and recurrence-free survival at pathological T1/2 and T3/4 in the matched cohort. **A** Overall survival and recurrence-free survival at pathological T1/2, **B** Overall survival and recurrence-free survival at pathological T3/4

After case matching, the 3-year OS and 3-year RFS rates were 86.5% and 77.9% in the CLS group and 98.4% and 88.5% in the RALS group, respectively. Although there were no significant differences in OS (*p* = 0.19) or RFS (*p* = 0.52) between the groups, the RALS group had slightly better OS and RFS rates **(**Fig. 1**)**. The OS and RFS at each stage in the matched cohort are shown in Fig. 2. The OS and RFS at pathological T1/2 and T3/4 in the matched cohort are shown in Fig. 3.

### Patterns of recurrence

**Fig. 4:**
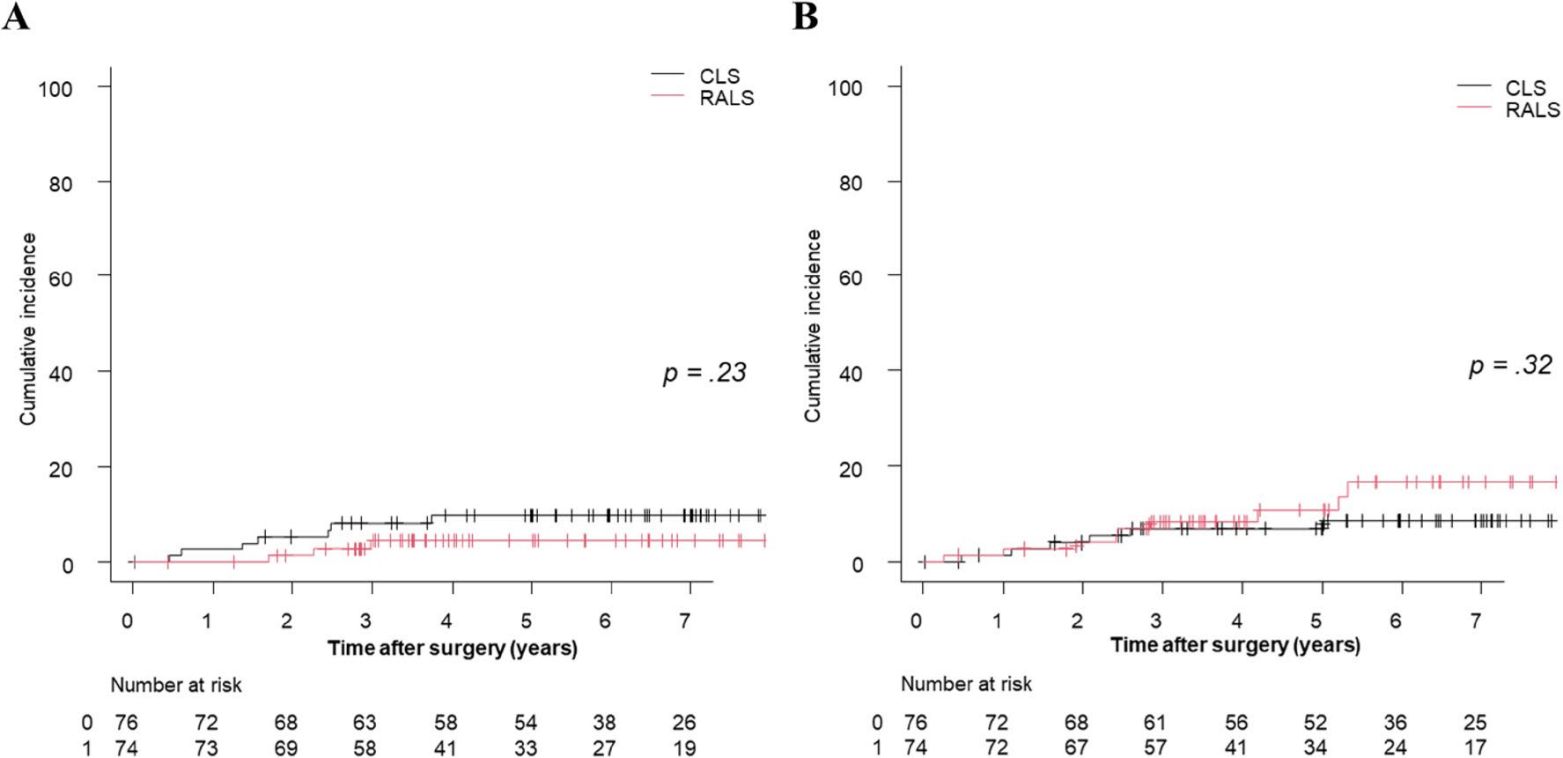
Cumulative local recurrence and distant metastasis rates by surgical procedure in the matched cohort. **A** Cumulative local recurrence rates, **B** Cumulative distant metastasis rates

Before case matching, the 3-year cumulative (Cum) LR and DM rates were 11.7% and 13.8% in the CLS group and 4.4% and 10.7% in the RALS group, respectively. There were no significant differences in Cum-LR (*p* = 0.08) or Cum-DM (*p* = 0.87) between the groups. After case matching, the 3-year Cum-LR and Cum-DM were 9.7% and 8.7% in the CLS group and 4.5% and 10.8% in the RALS group, respectively. There were no significant differences in Cum-LR (*p* = 0.23) or Cum-DM (*p* = 0.32) between the groups (Fig. 4).

## Discussion

Colorectal cancer is the second leading cause of cancer-related death in Japan. Surgical resection of colorectal cancer is the only curative treatment that is now increasingly performed using the robotic approach. However, its long-term oncological safety has not been fully verified. The present study aimed to retrospectively clarify the short- and long-term outcomes of RALS in consecutive patients with rectal cancer. In a minimal bias analysis with propensity score matching, the primary endpoint, 3-year RFS, was comparable to that reported previously in the RALS group [18, 19]. CLS and RALS showed comparable long-term oncologic outcomes in the present study. Furthermore, RLAS had better outcomes although the difference was not statistically significant. It is well known that the relapse of colon cancer is time-associated, with nearly 70% of recurrences occurring within the first two years after initial curative surgery [20]. Our primary endpoint was 3-year RFS, and a close look at the RFS survival curves in the matched cohort showed a large gap in survival rates between RALS group and CLS group around 3 years post-operatively. In the stage-by-stage analysis, the long-term results in the present study were comparable to those of known reports [15, 21]. Regardless of stage, there was no statistically significant difference in long-term oncologic outcomes between the two groups, but RALS tended to have better long-term results, suggesting the long-term oncological advantage of RALS over CLS. Furthermore, T4 rectal cancer is a challenging condition due to the highly invasive nature of the tumor, which can compromise curative resection. However, RALS for T4 rectal cancer has been reported to be associated with a significantly lower conversion rate, shorter hospital stay, and better long-term oncological outcomes than CLS [22]. In the present study, even in deeper tumor such as pathological T3/4, RLAS tended to have similar or better OS and RFS compared to CLS.

For rectal cancer, we focused on LR control by introducing neoadjuvant therapy and lateral lymph node dissection (LLND). However, DM occurs in approximately 30% of patients at 5 years after curative surgery [23]. In the present study, RALS did not contribute significantly to DM control; however, our findings suggest that it is associated with better local control than CLS. These long-term outcomes were similar to those reported previously [14, 20]. Concerns regarding laparoscopic rectal cancer surgery include technical limitations, such as a limited range of instrument movement and assistant-dependent camera control and traction, which are aggravated in the narrow surgical space during TME. However, currently available robotic surgical systems provide advanced technology and are used as an optional approach for TME. Robotic techniques have improved ergonomics, use articulated instruments, and provide a stable 3-dimensional view along with enhanced dexterity with tremor filtration and motion scaling. The superiority of local control by RALS, along with the development of distant control treatment with total neoadjuvant therapy, suggests the potential for improved outcomes in rectal cancer.

The short-term outcomes were not significantly different between the two groups after case matching. In the ROLARR randomized clinical trial, there was no significant difference in urinary dysfunction between the two groups. However, in the present study, IPSS scores were significantly better in the RALS group. These results are consistent with those reported in the literature [23, 24]. RALS with TME is associated with earlier recovery of normal urinary and sexual function than is CLS [6]. Further, the rate of urinary retention is also reportedly lower with RALS than with CLS or OS [7, 10]. A clear surgical field, in addition to high-quality three-dimensional imaging, may facilitate a decrease in the incidence of nerve injury, such as injury of the pelvic splanchnic nerves and inferior hypogastric plexus.

This study had some limitations. First, this was a single-center retrospective study. Second, although we performed propensity analyses to address this limitation, the study remains limited by the richness of the available data, and it might not be possible to make inferences due to hidden confounders. Fourteen covariates were included in the model used to calculate the propensity scores, but other covariates, such as symptoms, nutritional status, and social activity, were not included. Third, the sample size is relatively small. A prospective study is needed to adjust for other confounders and examine prognostic outcomes in our institute in future.

In conclusion, RALS is a reasonable option for the treatment of rectal cancer with long-term outcomes similar to those of CLS.

## Data Availability

The authors confirm that the data supporting the findings of this study are available within the article [and/or] its supplementary materials.
